# Supplementary material for: Suppressing hydrogen peroxide generation to achieve oxygen-insensitivity of a [NiFe] hydrogenase in redox active films
Source: Nat Commun. 2020 Feb 14;11:920. doi: 10.1038/s41467-020-14673-7 (PMC7021901; doi:10.1038/s41467-020-14673-7)
Supplement: Supplementary file 2 — Supplementary Information [file 41467_2020_14673_MOESM2_ESM.pdf]

Supplementary Information

Suppressing hydrogen peroxide generation to achieve oxygen-insensitivity of a [NiFe] hydrogenase in redox active films

Li *et al.*

Table of Contents:

Supplementary Figure 1: Effect of catalase in solution on film degradation during viologen catalyzed O_2 reduction.

Supplementary Figure 2: Effect of catalase in the film on film degradation during viologen catalyzed O_2 reduction.

Supplementary Figure 3: The effect of H_2O_2 added to the electrolyte on viologen polymer degradation.

Supplementary Note 1: Polymer film stability in presence of catalase and upon direct H_2O_2 addition.

Supplementary Figure 4: Normalized fluorescence emission spectra of the viologen moieties and of the H_2O_2 fluorescence reporter in absence and in presence of KI (0.1 M).

Supplementary Figure 5: Confocal fluorescence microscopy of viologen film and H_2O_2 detection for indicated time intervals.

Supplementary Figure 6: H_2O_2 detection at bare GCE using confocal fluorescence microscopy.

Supplementary Figure 7: CVs of viologen-modified polymer films on GC electrodes in anaerobic conditions.

Supplementary Figure 8: CVs of viologen-modified polymer films on GC electrodes in aerobic conditions.

Supplementary Figure 9: Reaction schematic for simulation the current response from catalysis in thin films.

Supplementary Note 2: Modeling and simulation description.

Supplementary Figure 10: Electrochemical O_2 reduction at bare GCE and polymer coated GCE.

Supplementary Figure 11: Catalytic current density versus H2 partial pressure in absence of O_2.

Supplementary Figure 12: Turnover stability of viologen-modified films containing hydrogenase.

Supplementary Figure 13: Biofuel cell replicate experiments.

Supplementary Figure 14: Effect of iodide on the catalytic activity of enzyme for H_2 oxidation.

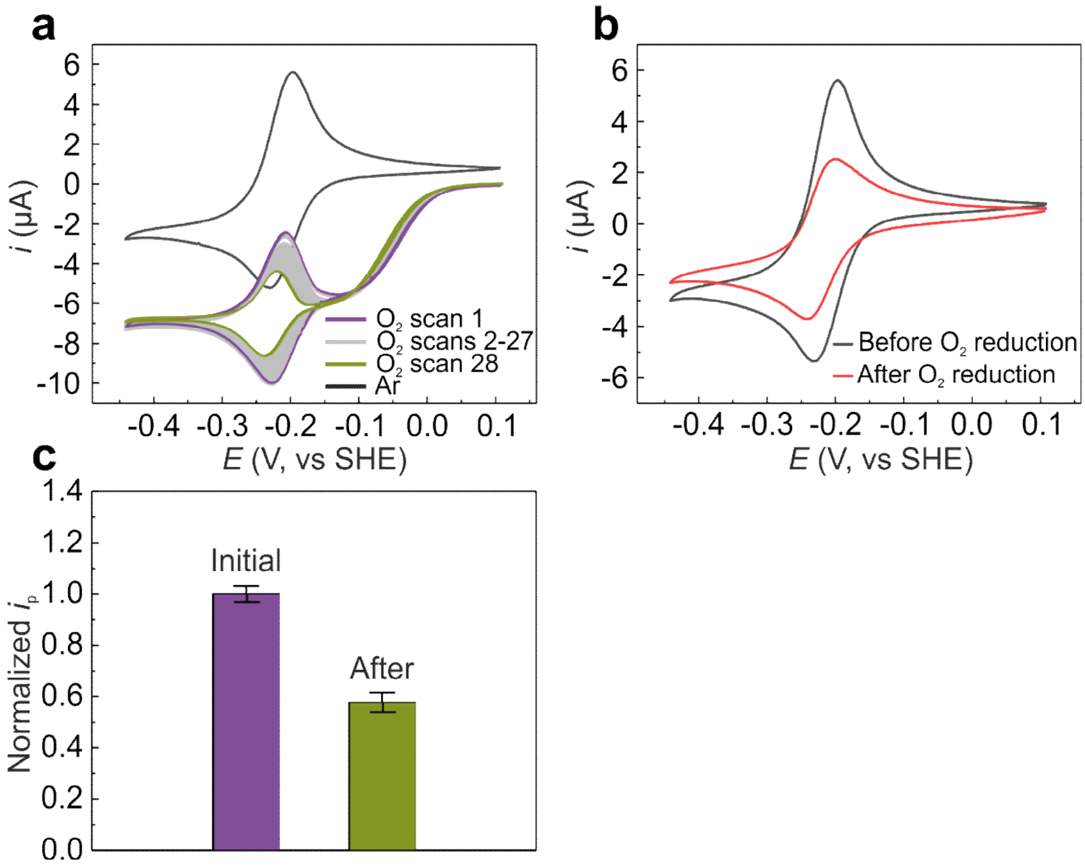

Supplementary Figure 1: **Effect of catalase in solution on film degradation during viologen catalyzed O_2 reduction** (**a**) Cyclic voltammograms (CVs, 28 cycles) of viologen-modified hydrogel films on glassy carbon electrodes under Ar and 5% O_2 in Ar. CV recorded under 100% Ar are shown as black curves. (**b**) CVs before (black) and after (red) cycling in presence of 5% O_2 in Ar. (**c**) Normalized peak current (i_p) before and after O_2 reduction derived from CVs under 100% argon. Error bars are defined as standard deviation. The currents and standard deviations are normalized by their average i_p before O_2 reduction. Average values and standard deviations were obtained from measurements of three individually prepared electrodes. All measurements were performed with GCE (3 mm in diameter) coated with viologen-modified polymer with a surface coverage of 0.3 mg cm^{-2}. Electrolyte: Phosphate buffer (0.1 M, pH 7) with 0.4 mg mL^{-1} catalase. Scan rate: 2 mV s^{-1}. Rotation rate: 2000 rpm. Source data are provided as a Source Data file.

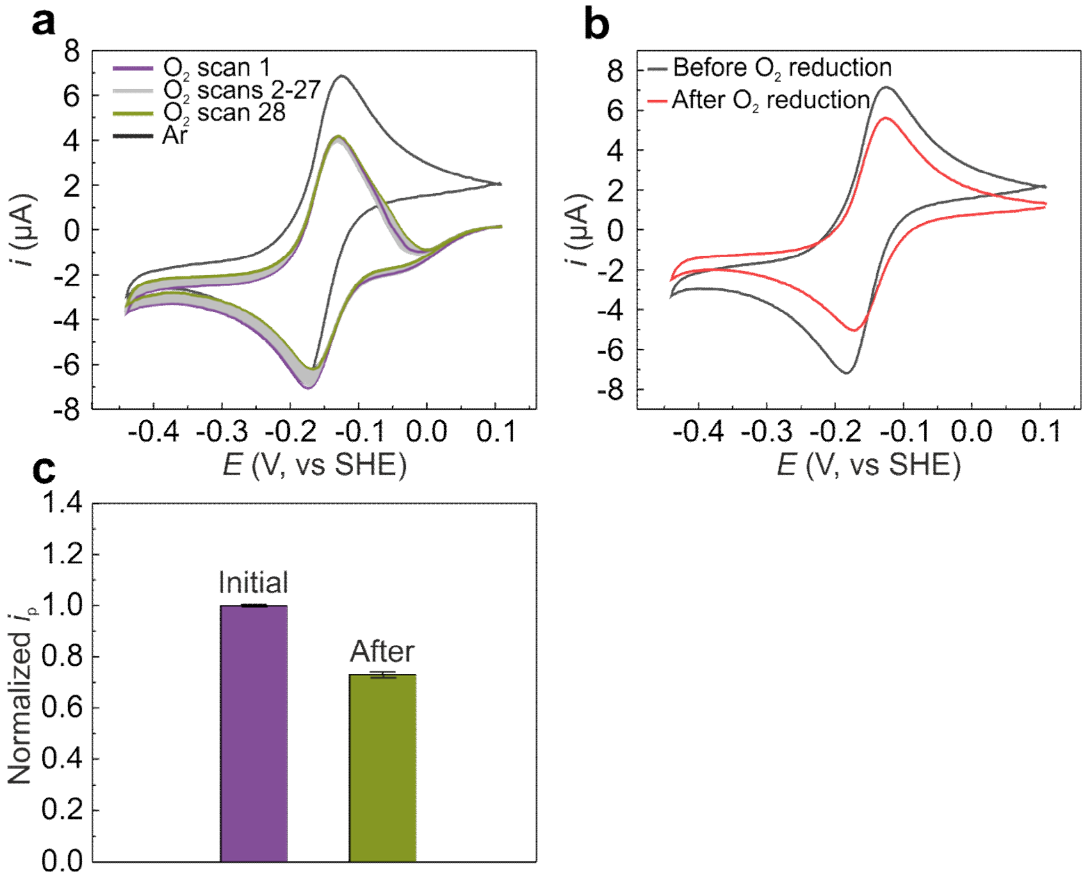

Supplementary Figure 2: **Effect of catalase in the film on film degradation during viologen catalyzed O_2 reduction** (**a**) Cyclic voltammograms (CVs, 28 cycles) of viologen-modified hydrogel films with catalase on glassy carbon electrodes under argon and 5% O_2 in Ar. CVs recorded under 100% Ar are shown in black. (**b**) CVs before (black) and after (red) cycling in presence of 5% O_2 in Ar. (**c**) Normalized peak current (i_p) before and after O2 reduction derived from CVs under 100% argon. The currents are normalized by their average i_p before O_2 reduction. Error bars are defined as standard deviation. Average values and standard deviations were obtained from measurements of three individually prepared electrodes. All measurements were performed with GCE (3 mm in diameter) coated with viologen-modified polymer with a surface coverage of 0.3 mg cm^{-2}. The loading of catalase in the film was 0.03 mg cm^{-2}. Electrolyte: Phosphate buffer (0.1 M, pH 7). Scan rate: 2 mV s^{-1}. Rotation rate: 2000 rpm. Source data are provided as a Source Data file.

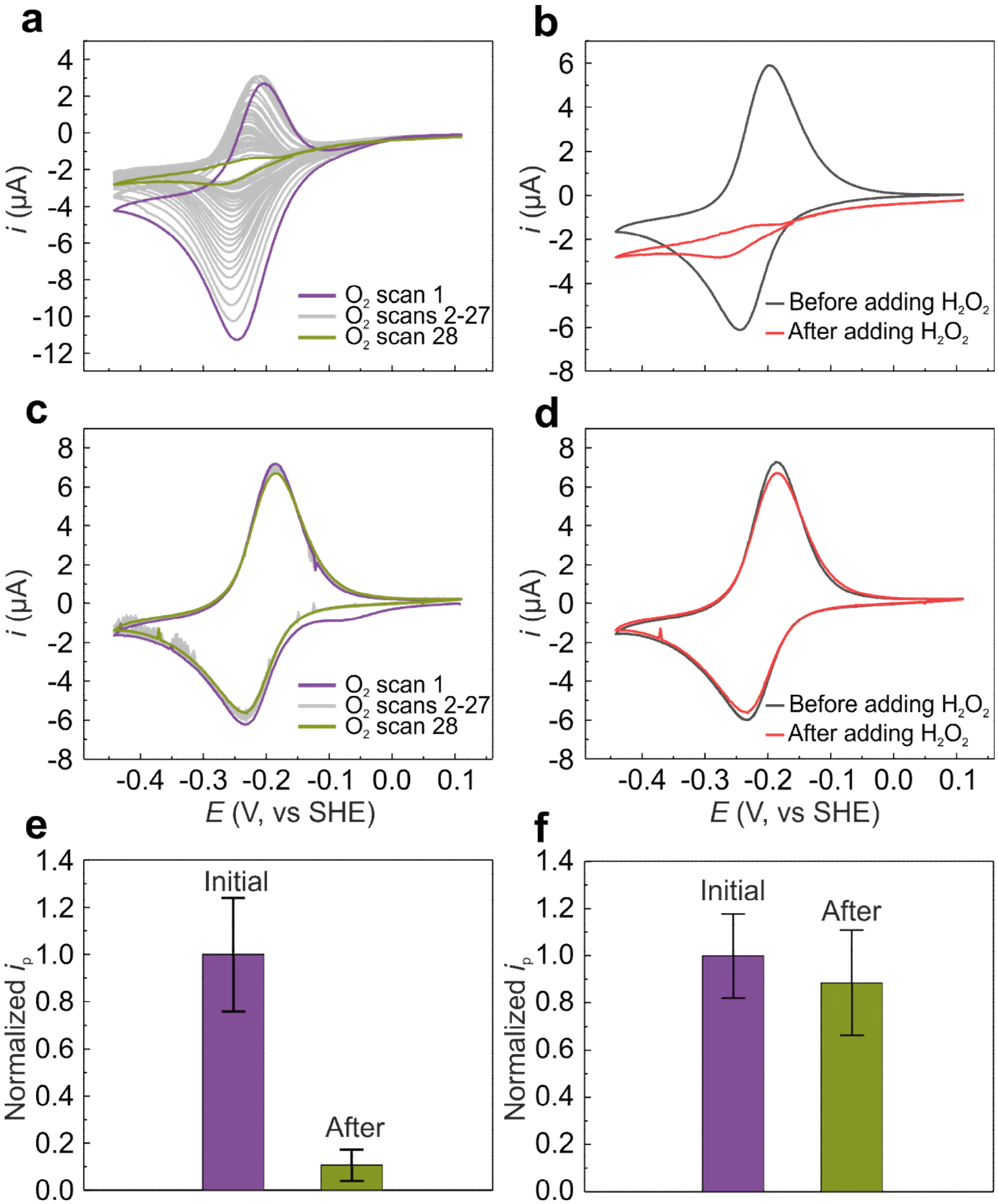

Supplementary Figure 3: The effect of H_2O_2 added to the electrolyte on viologen polymer degradation (**a**) 28 scans CVs of polymer film in PB containing H_2O_2 (10 mM), (**b**) CVs before adding H_2O_2 and after 28 scans with H_2O_2, (**c**) PB with H_2O_2 (10 mM) and catalase (0.4 mg mL^{-1}), (**d**) CVs before adding H_2O_2 and catalase and after 28 scans with H_2O_2 and catalase. Normalized peak current (i_p) (**e**) before and after adding H_2O_2 and (**f**) before and after adding H_2O_2 and catalase derived from CVs under 100% argon. The currents are normalized by their average i_p before adding H_2O_2. Error bars are defined as standard deviation. Average values and standard deviations were obtained from measurements of three individually prepared electrodes. All measurements were performed with GCE (3 mm in diameter) coated with polymer with a surface coverage of 0.3 mg cm^{-2}. Measurement conditions: Ar, electrolyte: Phosphate buffer (0.1 M, pH 7). The rotation rate was 2000 rpm. Scan rate: 2 mV s^{-1}. All measurements were performed at 298 K. Source data are provided as a Source Data file.

Supplementary Note 1: Polymer film stability in presence of catalase and upon direct H_2O_2 addition

We conducted two groups of control experiments to compare iodide and catalase for protecting the viologen modified film from H_2O_2 generated from viologen catalysed O_2 reduction.

First we tested the effect of employing catalase dissolved in the electrolyte. The concentration of catalase was set to 0.4 mg mL^{-1} because higher concentrations lead to excessive foam formation when purging the electrolyte with gases. Consecutive CV cycles of the polymer modified electrode in the presence of 5% O_2 in Ar showed a decrease of the peak current (**Supplementary Fig. 1a**). The peak current dropped to 56% of its original value after 4 hours of measurement (**Supplementary Fig. 1b, c**).

The catalase was subsequently immobilized in the film. In this case, consecutive CV cycles of the polymer modified electrode in the presence of 5% O_2 in Ar also showed a decrease of the peak current (**Supplementary Fig. 2a**). The peak current dropped to 70% of its original value after 4 hours of measurement (**Supplementary Fig. 2b, c**). This non-optimal protection from H_2O_2 by catalase is consistent with the previously reported low stability of hydrogenase in polymer films containing catalase[1]. In this previous report, the half-life in presence of catalase was less than 20 hours which is comparable to the values obtained without catalase for the system described in the present manuscript.

Moreover, H_2O_2 was added directly to the electrolyte to test its effect on the polymer film while cycling the potential between 0.1 and -0.45 V_{SHE}. After 28 consecutive CV cycles, the viologen signal is almost completely lost (**Supplementary Fig. 3a, b**). In contrast, if both catalase (0.4 mg mL^{-1}) and H_2O_2 (10 mM) are added to the electrolyte, the peak currents decreased by only 8% (**Supplementary Fig. 3c, d, e, f**). These results show that the viologen film is degraded by H_2O_2 and that catalase in solution efficiently removes H_2O_2 from the bulk electrolyte. However, it is not efficient for dismutating H_2O_2 when it is produced inside the hydrogel film (**Supplementary Figs. 1 and 2**).

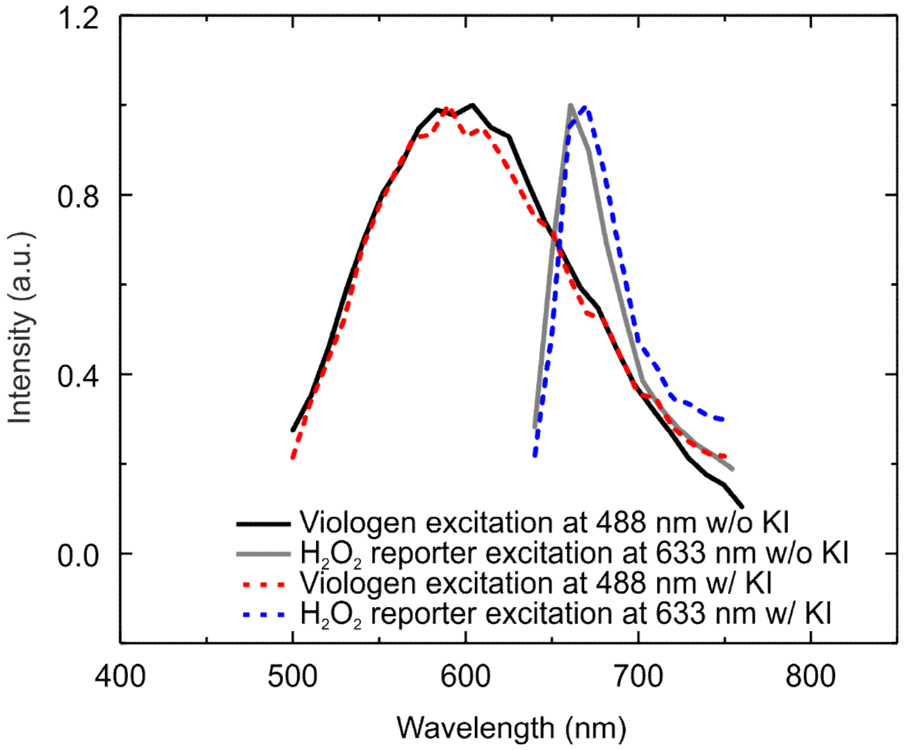

Supplementary Figure 4: Normalized fluorescence emission spectra of the viologen moieties and of the H_2O_2 fluorescence reporter in absence and in presence of KI (0.1 M) The emission spectra of viologen moieties excited at 488 nm are shown in black (w/o KI) and red (w/ KI), respectively. For the oxidized form of the H_2O_2 probe excited at 633 nm, the spectra are shown in grey (w/o KI) and dashed blue (w/ KI), respectively. Spectra were recorded in presence of O_2 with an applied potential of -0.1 V vs SHE at the electrode. Electrolyte: Phosphate buffer (0.1 M, pH 7). Surface coverage of the polymer on the electrode is 2.26 mg cm^{-2}.

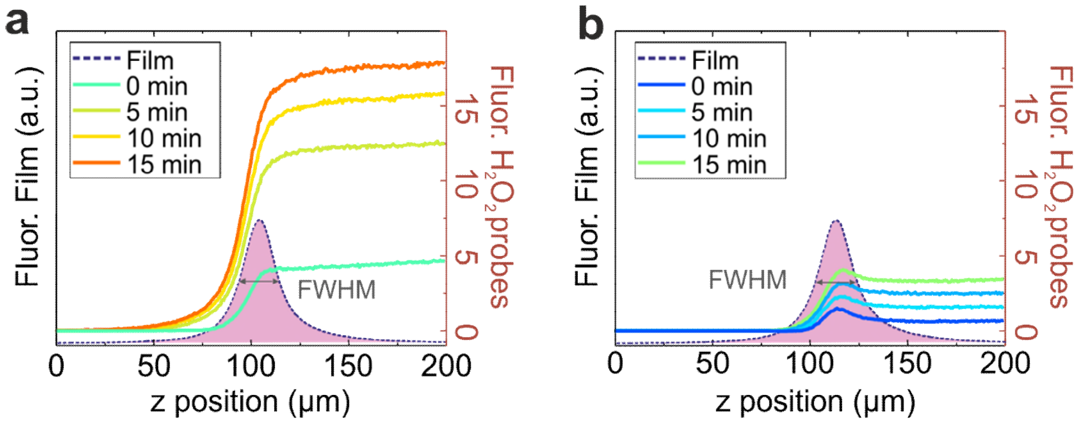

Supplementary Figure 5: Confocal fluorescence microscopy of viologen film and H_2O_2 detection for indicated time intervals (**a**) w/o KI and (**b**) w/ KI in electrolyte. The fluorescence of the film was derived from the viologen moieties in the oxidized state (at open circuit potential in air). Starting from 0 min, a constant potential at -0.1 V_{SHE} was applied to the electrodes. The measurements were performed with GCE (3 mm in diameter) in phosphate buffer (0.1 M, pH 7). The fluorescence of the oxidized viologen-modified films was collected around its maximum emission of 590 nm upon excitation at 488 nm. The emission of the H_2O_2 fluorescent probe was determined around its maximum emission of 660 nm upon excitation at 633 nm. Measurements were performed under ambient air at 300 K.

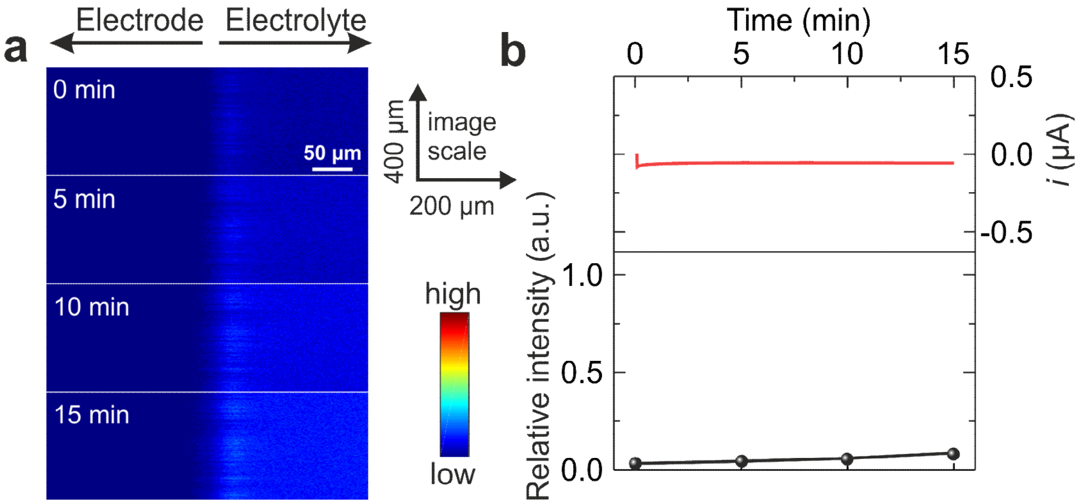

Supplementary Figure 6: H_2O_2 detection at bare GCE using confocal fluorescence microscopy (**a**) Representative fluorescent depth profile images of H_2O_2 sensitive indicators (0 min – 15 min) at bare GCE. (**b**) O_2-reduction current on bare GCE at a constant potential of -0.1 V *vs* SHE and the relative intensity of fluorescent H_2O_2 reporter derived from (**a**) normalized by the maximum value observed in Fig. 2. The measurements were performed with GCE (3 mm in diameter) in phosphate buffer (0.1 M, pH 7). Emission of the H_2O_2 fluorescent probe was determined around its maximum emission of 660 nm upon excitation at 633 nm. The scale is the same for all images. Measurements were performed under ambient air at 300 K.

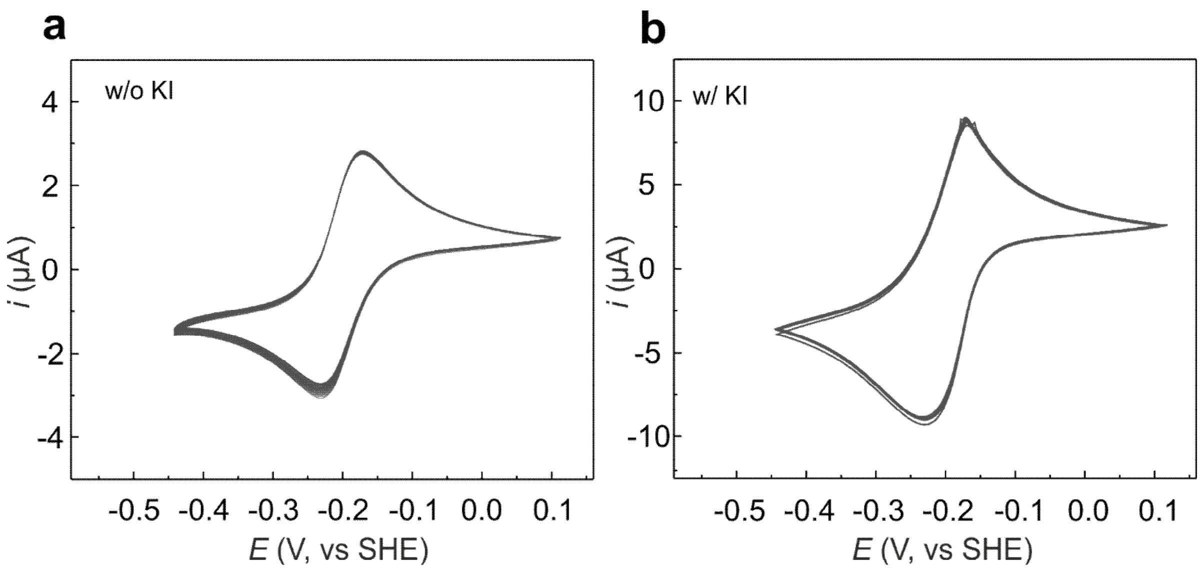

Supplementary Figure 7: CVs of viologen-modified polymer films on GC electrodes in anaerobic conditions (**a**) Continuous 28 CVs in absence of KI. The average peak currents from three individually prepared electrodes was 3.3 ± 0.6 μA which decreased by about 0.08 ± 0.3 μA after 28 cycles (about 2.4 % decrease) (**b**) Continuous 28 CVs in presence of KI (0.1 M) in the electrolyte. The average peak currents from three individually prepared electrodes was 8.2 ± 1.2 μA which decrease by about 0.17 ± 0.4 μA after 28 cycles (about 2.1 % decrease). All measurements were performed under 100 % Ar at 298 K in phosphate buffer (0.1 M, pH 7) at a scan rate of 2 mV s^{-1}. Rotation rate: 2000 rpm. Surface coverage of the polymer on the electrode was 0.3 mg cm^{-2}. The peak currents in presence of KI are typically higher compared to the measurement carried out in pure PB most likely because iodide as a hydrophobic counter-ion partially desolvates the film which enhances the concentration of the viologen moieties and electron hopping rates.[2,3] Source data are provided as a Source Data file.

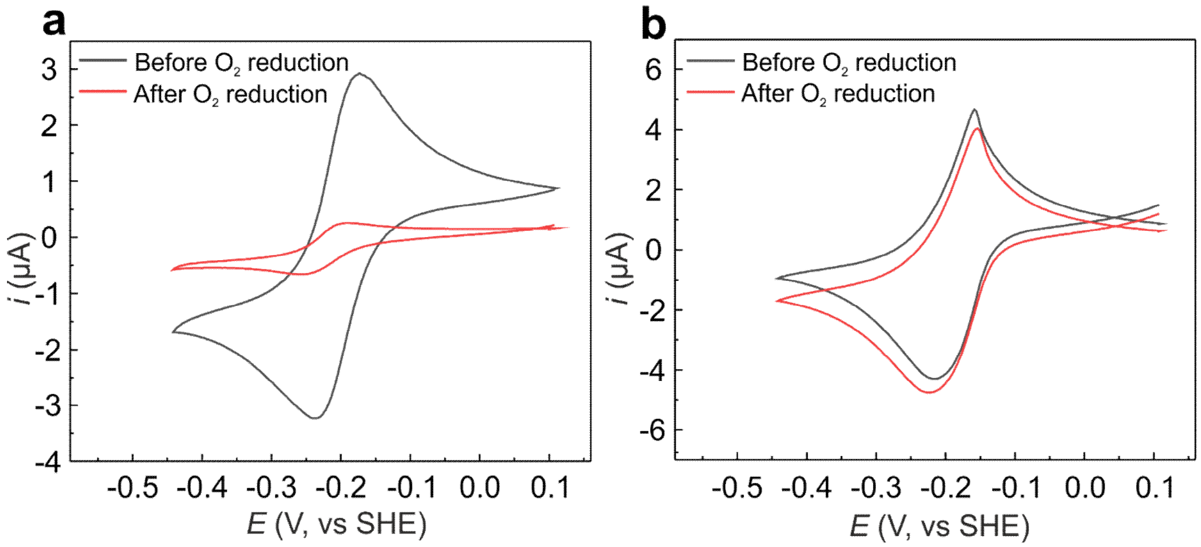

Supplementary Figure 8: Comparison of CVs of viologen-modified polymer films on GC electrodes in anaerobic conditions before and after O_2 reduction CVs before (black) and after (red) cycling in presence of 5% O_2 in Ar (see Fig. 3) (**a**) in absence and (**b**) in presence of KI (0.1M) in the electrolyte. All measurements were performed under 100 % Ar at 298 K in phosphate buffer (0.1 M, pH 7) at a scan rate of 2 mV s^{-1}. Rotation rate: 2000 rpm. Surface coverage of the polymer on the electrode was 0.3 mg cm^{-2}. The peak currents in presence of KI are typically higher compared to the measurement carried out in pure PB buffer most likely because iodide as a hydrophobic counter-ion partially desolvates the film which enhances the concentration of the viologen moieties and electron hopping rates.[2,3] Source data are provided as a Source Data file.

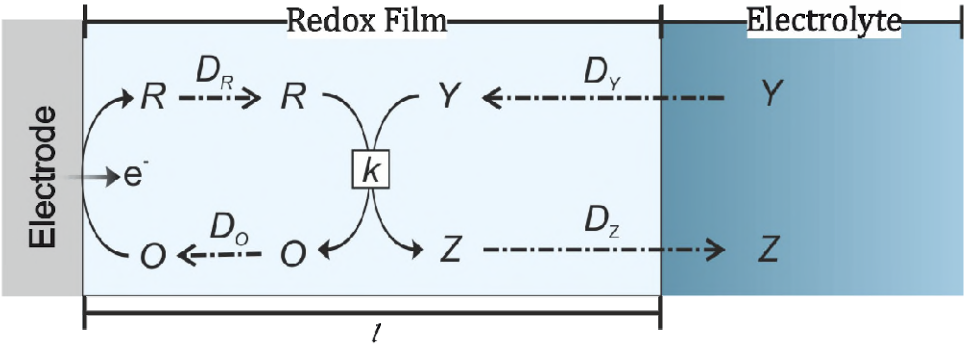

Supplementary Figure 9: Reaction schematic for simulation the current response from catalysis in thin films Within a film of length *l* which is drop-cast onto an electrode with surface area *A*, the redox active viologen moieties (concentrations C_O and C_R, for the oxidized and reduced states respectively, total concentration O_{Tot}) are confined to the film and are assumed to undergo rapid heterogeneous electron transfer kinetics at the electrode interface. At the beginning of the experiment, all viologen moieties are assumed to be in the oxidized state. As the potential is linearly cycled between the initial and final potential values, *R* is produced at the electrode surface. As *R* diffuses (apparent diffusion with a diffusion coefficient D_R) through the film by means of electron hopping, it irreversibly reacts (second order reaction rate constant *k*) with reactant *Y*, generating *Z* and regenerating *O*, which also diffuses through the film (apparent diffusion via electron hopping) with a diffusion coefficient D_o. In contrast to *O* and *R*, *Y* and Z are freely diffusing (diffusion constant D_Y and D_Z respectively) both within the film and in the surrounding solution. Due to the rapid rotation of the external solution, the concentration of *Y* at the film/solution interface remains constant at its total value (Y_{Tot}), which is equal to its concentration in the surrounding solution, which is also uniform. Within the film, however, the concentration of *Y* can vary with position.

Supplementary Note 2: Modeling and Simulation Description

A comprehensive model for stationary cyclic voltammetry of electrodes modified with redox active films was reported.[4] In addition, this model was further developed for the case of fast catalysis where substrate concentration in the film is constant[5] (depletion of the substrate concentration in the film is not allowed). We developed a related model (Supplementary Figure 9) because the previously reported models are not directly applicable for our experimental system, in which there is rapid rotation of the electrode and possibly depletion of the substrate (oxygen) within the redox active film.

The modeling equations consist of material balances on *O* and *Y* within the redox film (Supplementary Equation 1 and 2 respectively), resulting in a system of two partial differential equations (PDEs) that must be solved simultaneously for the time and space dependent concentration profiles of *O* and *Y*.

$$\frac{\partial C_O}{\partial t} = D_O \frac{\partial^2 C_O}{\partial x^2} + k(O_{Tot} - C_O)(C_Y)$$

(Supplementary Equation 1)

$$\frac{\partial C_Y}{\partial t} = D_Y \frac{\partial^2 C_Y}{\partial x^2} - k(O_{Tot} - C_O)(C_Y)$$

(Supplementary Equation 2)

The current is calculated based on the time dependent concentration gradient of *O* at the electrode surface (Supplementary Equation 3).

$$I = -nFAD_O \left(\frac{\partial C_O}{\partial x}\right)_{x=0}$$

(Supplementary Equation 3)

At the initial condition, C_O and C_Y are equal to their total concentrations (O_{Tot} and Y_{Tot}), respectively. The electron transfer at the electrode surface is rapid and is modeled according to Butler-Volmer kinetics; with respect to *Y*, the electrode is inert and impermeable. At the film/solution boundary, the flux of *O* and *R* out of the film is zero since it is confined, and the concentration of *O* is equal to Y_{Tot} because of perfect mixing in the external solution as a result of the high rotation speed.

Before solving numerically, the time, space, and concentration variables were all scaled with respect to their maximum values. The system was numerically solved by means of the method of lines, in which the space variable was discretized, resulting in an equivalent system of simultaneous ordinary differential equations (ODEs). The set of finite difference ODEs were individually derived by material balances within the discretized control volumes (finite volume method). The ODE system was solved numerically using the Julia programming language, by making use of the LSODA solver within the DifferentialEquations.jl package.

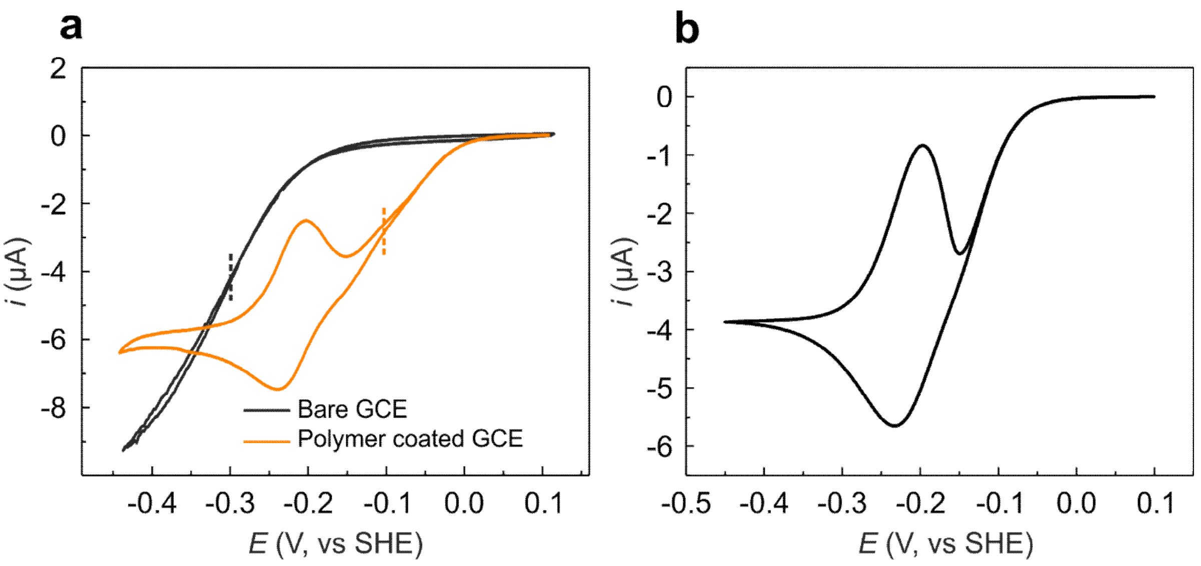

Supplementary Figure 10: Electrochemical O_2 reduction at bare GCE and polymer coated GCE (**a**) CVs of viologen-modified hydrogel films on GCE (orange) and bare GCE (black) under 5% O_2 in Ar. Both measurements were performed at 298 K in phosphate buffer (0.1 M, pH 7) at a scan rate of 10 mV s^{-1} with a rotation rate of 2000 rpm. Surface coverage of the polymer on the electrode is 0.3 mg cm^{-2}. (**b**) Simulation results for polymer coated electrodes based on the kinetic scheme in Supplementary Fig. 9 with the following parameter values: l = 10 µm, A = 0.071 cm^2, O_{Tot} = 60 mM, Y_{Tot} = 0.05 mM, k = 1 x 10^6 M^{-1} s^{-1}, E^0 = -0.210 V, scan rate = 0.10 V s^{-1}, D_O = D_R = 1.0 x 10^{-9} cm^2 s^{-1} and D_Y = D_Z = 1.0 x 10^{-5} cm^2 s^{-1}.

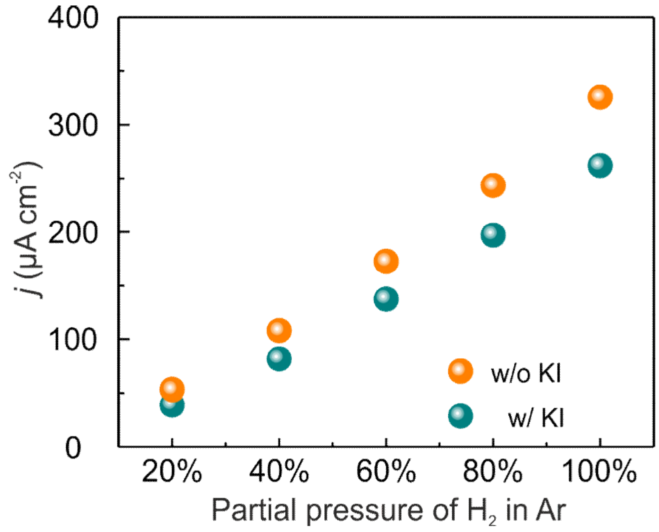

Supplementary Figure 11: Catalytic current density versus H_2 partial pressure in absence of O_2 Catalytic current from the polymer-hydrogenase electrode in absence and in presence of KI (0.1M). All measurements were performed in phosphate buffer (0.1 M, pH 7) at an applied potential of 0.21 V *vs* SHE with a rotation rate of 2000 rpm at 298 K. Surface coverage of the polymer on the electrode was 2.26 mg cm^{-2}.

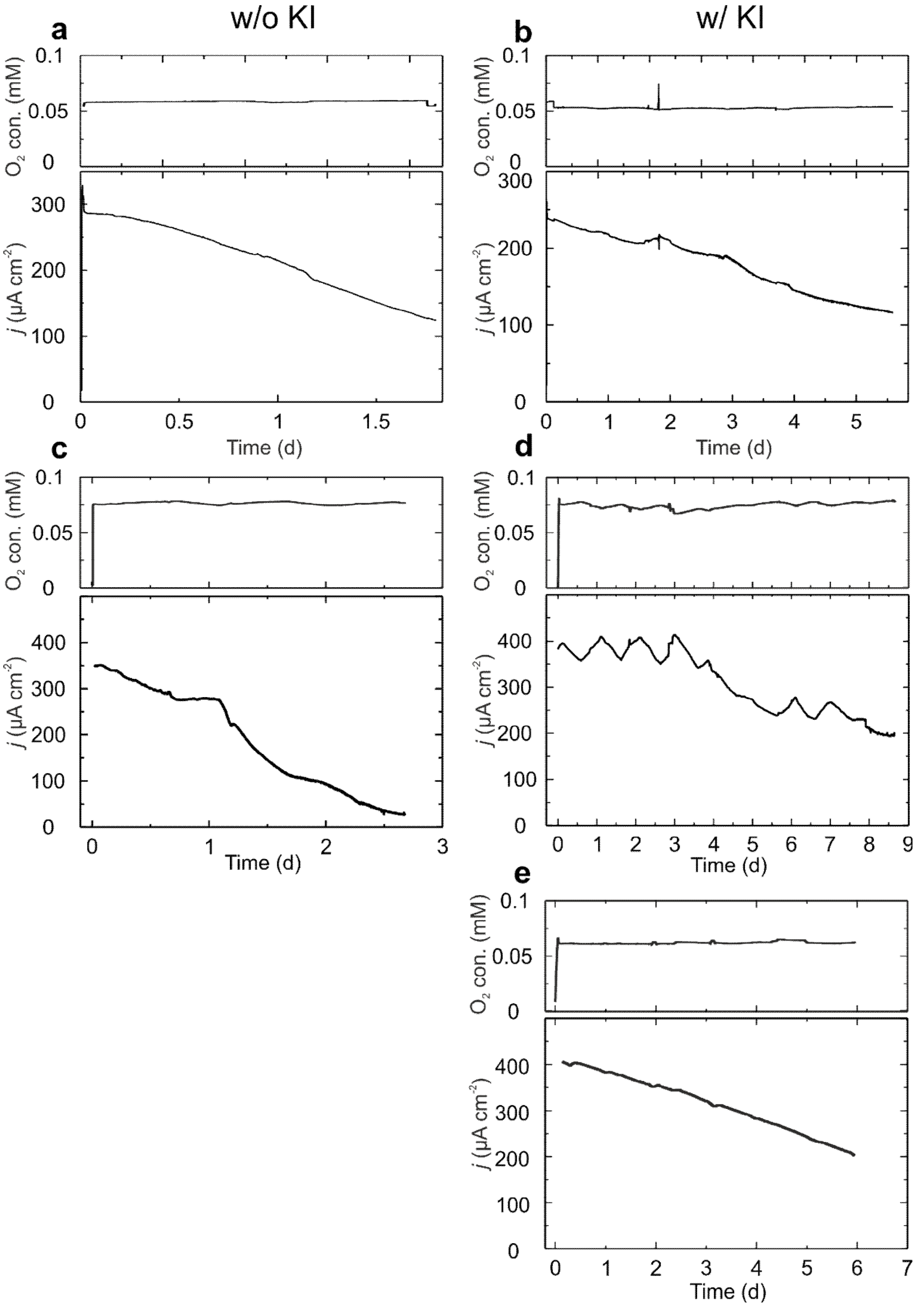

Supplementary Figure 12: Turnover stability of viologen-modified films containing hydrogenase Catalytic current from the polymer-hydrogenase electrode (**a**), (**c**) in the absence and (**b**), (**d**), (**e**) in the presence of KI (0.1M). The measurements (**a**), (**b**), (**c**) and (**d**) were conducted at 298 K and (**e**) at 333K under aerobic conditions (5% O_2 in Ar). All measurements were performed in phosphate buffer (0.1 M, pH 7) at an applied potential of 0.21 V *vs* SHE with a rotation rate of 2000 rpm. Surface coverage of the polymer on the electrode was 2.26 mg cm^{-2}.

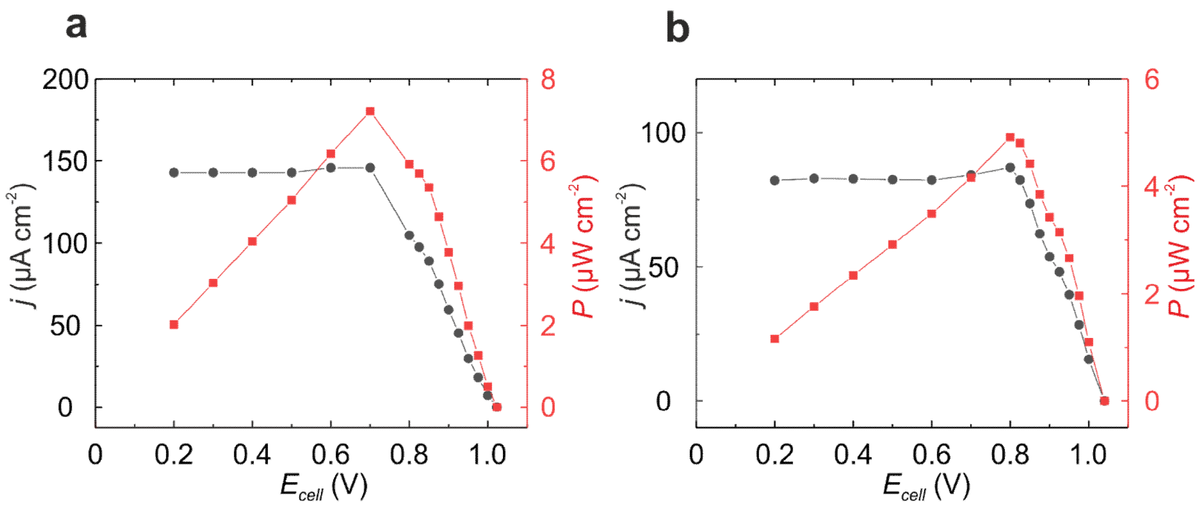

Supplementary Figure 13: Biofuel cell replicate experiments (**a**) First replicate and (**b**) second replicate. Current density (black solid dots) and power density (red solid squares) versus cell voltage. Fuel cell measurements were conducted in a two compartment cell with a glass frit as separator. The anode modified with hydrogenase and the viologen polymer was placed in the compartment containing phosphate buffer (0.1 M, pH 7) and KI (0.1 M) purged with 100% H_2. The cathode modified with bilirubin oxidase for O_2 reduction was placed in the compartment containing phosphate buffer (0.1 M, pH 7) purged with 100% O_2. The polymer surface coverage for all hydrogenase modified electrodes is 2.26 mg cm^{-2}. All measurements were performed at 298 K.

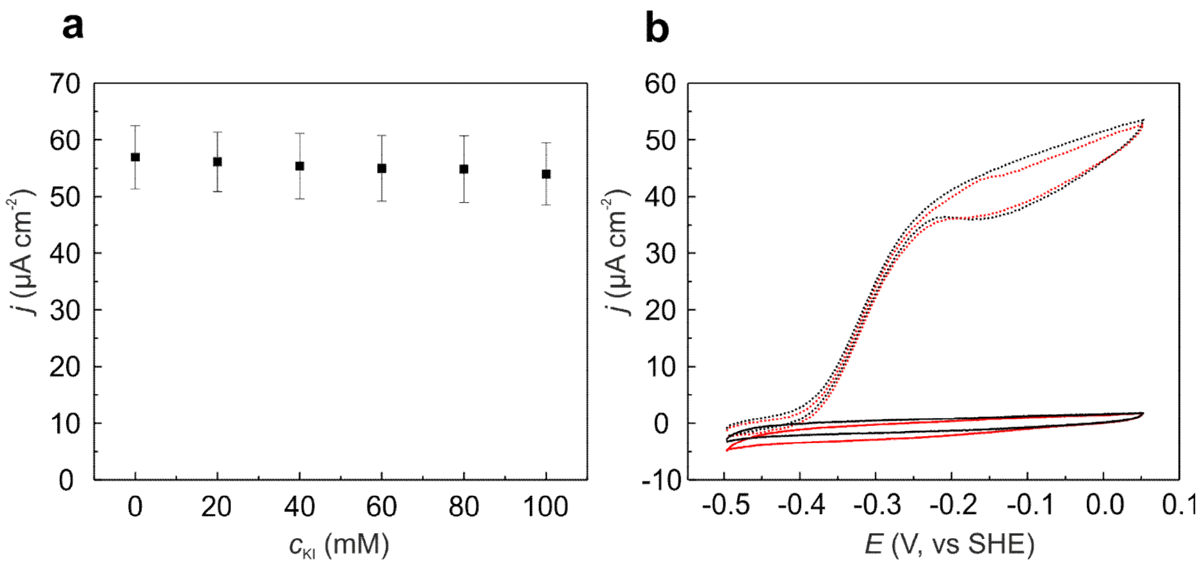

Supplementary Figure 14: Effect of iodide on the catalytic activity of enzyme for H_2 oxidation (a) Titration of the buffer solution with concentrations of KI of 20, 40, 60, 80 and 100 mM. Error bars are defined as standard deviation. Three replicates with individually prepared electrodes were performed for each concentration; the current was recorded at -150 mV (*vs* Ag/AgCl 3M KCl) and plotted against KI concentrations. **(b)** Cyclic voltammetry experiments of pyrolytic graphite electrodes without enzyme (solid line) and covalently modified with hydrogenase in direct electron transfer configuration (dotted lines) with the addition of 0.1 M KI (red traces) and without KI (black traces). Scan rate: 10 mV s^{-1}. Electrolyte: phosphate buffer (0.1 M, pH 7), 100% H_2. *Dv*MF [NiFe] hydrogenase was immobilized on a pyrolytic graphite electrode (PGE) using a modified procedure from references 6 and 7. PGE was first polished with abrasive paper (P1000) then with alumina slurry (1 µm) and sonicated in water for 10 min. Afterwards, the polished PGE was immersed in a 10 mM solution of 4-nitrobenzene diazonium tetrafluoroborate salt 96% (Sigma-Aldrich) in acetonitrile (Merck) for 10 min. After rinsing the electrode with ethanol and water, the immobilized 4-nitrobenzene molecules were electrochemically reduced to 4-aminobenzene in 0.1 M H_2SO_4 by means of cyclic voltammetry from 0.6 V to -1 V (vs Ag/AgCl) at 0.1 V/s. Afterwards 2 µL of 200 µM *Dv*MF hydrogenase in 10 mM MES buffer (pH 5.8) were placed on top of the electrode for 20 min, then 3 µL N-hydroxysuccinimide (Sigma) and 3 µL N-(3-dimethylaminopropyl)-N'-ethylcarbodiimide hydrochloride (Sigma) 60 mM each were added and left to react for 1 h. Afterwards the electrode was rinsed with water and placed on the electrochemical cell for measurements. Source data are provided as a Source Data file.

Supplementary References

1. Ruff, A. *et al.* A fully protected hydrogenase/polymer-based bioanode for high-performance hydrogen/glucose biofuel cells. *Nat. Commun.* **9,** 3675 (2018).
2. Kothe, T. *et al.* Engineered electron-transfer chain in photosystem 1 based photocathodes outperforms electron-transfer rates in natural photosynthesis. *Chem. Eur. J.* **20,** 11029–11034 (2014).
3. Blauch, D. N. & Savéant, J. M. Dynamics of electron hopping in assemblies of redox centers. Percolation and diffusion. *J. Am. Chem. Soc.* **114,** 3323–3332 (1992).
4. Costentin, C., Savéant, J.M., Cyclic Voltammetry Analysis of Electrocatalytic Films. *J. Phys. Chem. C.* **119**, 12174-12182 (2015).
5. Costentin, C., Savéant, J.M., Cyclic Voltammetry of Electrocatalytic Film: Fast Catalysis Regimes. *ChemElectroChem* **2**, 1774-1784 (2015).
6. Plumeré, N. *et al.* A redox hydrogel protects hydrogenase from high-potential deactivation and oxygen damage. *Nat. Chem.* **6,** 822–827 (2014).
7. Rüdiger, O., Abad, J. M., Hatchikian, E. C., Fernandez, V. M. & Lacey, A. L. de. Oriented immobilization of Desulfovibrio gigas hydrogenase onto carbon electrodes by covalent bonds for nonmediated oxidation of H_2. *J. Am. Chem. Soc.* **127,** 16008–16009 (2005).
