# Supplementary material for: Suppressing hydrogen peroxide generation to achieve oxygen-insensitivity of a [NiFe] hydrogenase in redox active films
Source: Nat Commun. 2020 Feb 14;11:920. doi: 10.1038/s41467-020-14673-7 (PMC7021901; doi:10.1038/s41467-020-14673-7)
Supplement: Supplementary file 3 — Description of Additional Supplementary Files [file 41467_2020_14673_MOESM3_ESM.pdf]

Description of Additional Supplementary Files

1. File Name: Supplementary Movie 1

   Description: CARS signal of viologen-modified film in the presence of KI.

2. File Name: Supplementary Movie 2

   Description: CARS signal of viologen-modified film in the absence of KI.

3. File Name: Supplementary Movie 3

   Description: Fluorescence of viologen-modified film in the presence of KI.

4. File Name: Supplementary Movie 4

   Description: Fluorescence of viologen-modified film in the absence of KI.
